# Combination of *in vivo* and *in vitro* phosphoproteomics determines the PP2A target repertoire on proteome scale

**DOI:** 10.1016/j.crmeth.2025.101084

**Published:** 2025-06-19

**Authors:** Melanie Brunner, Zehan Hu, Heidy Elkhaligy, Gloria Lampo, Carole Roubaty, Christine Vionnet, Devanarayanan Siva Sankar, Sean J. McIlwain, Stéphanie Kaeser-Pebernard, Yongna Xing, Jörn Dengjel

**Affiliations:** 1 Department of Biology, University of Fribourg, Chemin du Musée 10, 1700 Fribourg, Switzerland; 2 Biophysics Program, University of Wisconsin at Madison, Madison, WI 53706, USA; 3 Biostatistics and Medical Informatics, Wisconsin Institutes of Medical Research, University of Wisconsin at Madison, School of Medicine and Public Health, Madison, WI 53705, USA; 4 Department of Oncology, University of Wisconsin at Madison, School of Medicine and Public Health, McArdle Building on Lorch Street, Madison, WI 53706, USA

**Keywords:** CAPRIN1, mass spectrometry, kinase, phosphatase, PP1, PP2A, PPP2R5E, proteomics, signaling, stress granule

## Abstract

Dynamics of protein phosphorylation are regulated by the interplay of kinases and phosphatases. Current mass spectrometry-based phosphoproteomic approaches are extremely powerful in identifying and quantifying tens of thousands of phosphosites in single biological samples. However, whereas the mapping of phosphosites is successfully automated supporting high sample throughput, the characterization of responsible kinases and phosphatases still largely depends on laborious protein biochemical assays. To show direct (de)phosphorylation events, *in vitro* kinase or phosphatase assays using single substrates or peptide arrays are often used. Here, we describe the development of an *in vitro* phosphatase assay using whole proteome under native conditions as input. We employ this approach to study the PP1 and PP2A target repertoire, characterizing thousands of potential target sites. Focusing on PPP2R5E/B56ε-containing complexes, we combine *in vitro* with *in vivo* phosphoproteomics to characterize bona fide target sites, which highlight the role of PP2A in regulating stress granule assembly.

## Highlights

• Whole-proteome *in vitro* phosphatase assay supports phosphatase-target site screening
• *In vitro* and *in vivo* phosphoproteomics shortlist numerous bona fide PP2A target sites
• PP2A complexes target phosphosites on protein translation machinery
• PPP2R5E/B56ε-containing complexes negatively regulate stress granule formation

## Motivation

*In vitro* assays remain the gold standard for proving direct phosphatase-substrate interactions. Performing such assays on proteome scale under native conditions—i.e., retaining the enzymatic activities of endogenous protein complexes—is challenging, as primary and secondary effects are difficult to discriminate. We developed an on-bead *in vitro* phosphatase assay that supports the inactivation of endogenous phosphatases and the analysis of exogenous phosphatase-substrate interactions on proteome scale. By coupling this assay with quantitative phosphoproteomics, we screen for phosphatase-substrate interactions, identifying bona fide phosphatase target sites in high throughput.

## Graphical abstract

**Figure:**
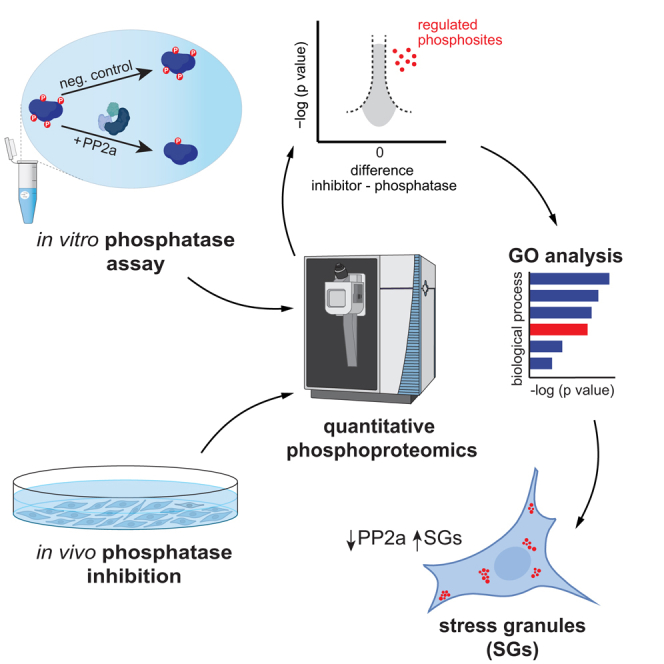

## Teaser

Brunner et al. developed an on-bead *in vitro* phosphatase screen that uses whole-proteome lysates as input. In combination with *in vivo* phosphoproteomics, they outline the PP2A target repertoire. Focusing on PPP2R5E/B56ε-containing PP2A complexes, they highlight that PP2A negatively affects stress granule formation.

## Introduction

Phosphorylation-based signaling is a dynamic process essential for regulating key cellular functions. It is governed by the interplay of kinases and phosphatases,^1^ its dysregulation being linked to human diseases.^2^ Compared to kinases, phosphatases are understudied,^3^^,^^4^ which is likely based on the misconception that phosphatases are rather unspecific, and thus not attractive drug targets. As the human genome encodes 518 kinases but only 189 phosphatases, 105 of which have been shown to be catalytically active,^5^^,^^6^ it was thought that phosphatase inhibition/activation does not interfere with specific cellular pathways but has non-specific effects affecting metabolism as a whole. In the last few years, however, this view has changed dramatically.^3^ Protein phosphatases commonly do not exist as free catalytic subunits, but are part of multiprotein complexes forming holoenzymes in which a catalytic subunit is bound to one or more scaffold and regulatory subunits, which determine subcellular localization and substrate specificity.^7^ While protein kinase specificity heavily relies on the recognition of specific consensus sequences surrounding the phosphorylation sites, protein phosphatases seem to lack a discernible consensus motif. Recent work has demonstrated that catalytic and regulatory subunits can bind to short linear motifs (SLiMs), with specific consensus sequences tailored to each phosphatase.^7^^,^^8^^,^^9^ These SLiMS are short amino acid sequences that reside primarily in disordered regions of substrates and are spatially separated from the actual dephosphorylation sites. Next to SLiMS, secondary structural elements like α helices can promote phosphatase-target protein interactions.^10^

With respect to serine- and threonine-phosphorylation, which, depending on the experimental setting, accounts for ca. 90% of observed protein phosphorylation events in mammalian cells,^11^ more than 90% of dephosphorylation events are believed to be orchestrated by the seven members of the protein phosphatase family (PPP), PP1–PP7.^12^ Within this family, PP1 and PP2A are collectively responsible for dephosphorylating the majority of serine/threonine phosphorylation sites.^13^ Both phosphatases form protein complexes involving catalytic and scaffold subunits that facilitate substrate binding. It is believed that several tens to hundreds of different PP1 and PP2A holocomplexes exist in human cells, regulatory subunits conferring localization and specificity.^3^^,^^4^

While there have been substantial advancements in phosphatase research methodologies,^14^ there is still a need for more comprehensive approaches.^15^ In particular, the majority of mass spectrometry (MS)-based phosphoproteomic studies, which enable site-specific identification and quantification of tens of thousands of phosphosites in single analyses, have focused on kinases.^16^ In contrast, only a few studies specifically address phosphatase targets.^14^^,^^17^^,^^18^ To support the unbiased analysis of direct phosphatase targets, we developed an on-bead *in vitro* phosphatase assay (OBIPhA) that uses whole proteome as input and thus supports screening approaches. The assay is based on the recently developed on-bead *in vitro* kinase assay (OBIKA) preserving protein-protein interactions and maintaining quarternary structures.^19^ This approach offers distinct advantages over conventional methods that typically involve working with denatured proteins or peptide arrays. By retaining the native status of proteins, this assay enables *in vitro* monitoring of dephosphorylation events under physiologically relevant conditions. In the present paper, we employed this assay to study PP1 and PP2A targets, focusing on the latter. We expand the PP2A target repertoire specifically of PPP2R5E/B56ε-containing holoenzymes, emphasizing their role in stress granule assembly.

## Results and discussion

### Whole-proteome OBIPhA

**Figure 1:**
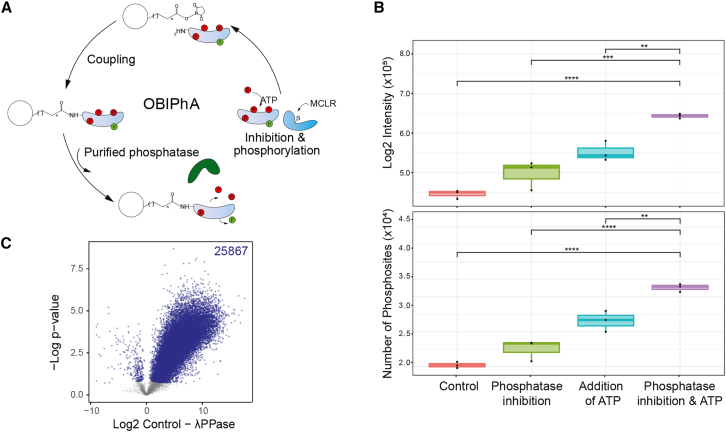
Workflow and experimental results for proteome-wide on-bead *in vitro* phosphatase assay (A) On-bead *in vitro* phosphatase assay (OBIPhA) workflow. After the indicated dephosphorylation of target sites, the samples were prepared for bottom-up phosphoproteomic analysis. (B) Immobilized endogenous kinases and phosphatases are still active, as indicated by the intensities and numbers of identified phosphosites in the respective experimental conditions. Box plots show quantifications of *n* = 3 biological replicates (black dots), boxes are drawn according to ggplot2 standard settings. ∗∗*p* < 0.01; ∗∗∗*p* < 0.001; ∗∗∗∗*p* < 0.0001; Student’s t test, unpaired. (C) Exogenous addition of λ-phosphatase leads to a significant decrease in phosphorylation sites. Volcano plot indicates broad dephosphorylation of immobilized proteome, with 25,867 significantly reduced phosphorylation sites compared to control (*n* = 3 biological replicates, FDR <0.05, Student’s t test, unpaired). Significantly changing sites are marked in blue. See also Table S1.

To interrogate direct dephosphorylation events while discriminating them from indirect, secondary effects by MS-based phosphoproteomics, we developed OBIPhA. This method is based on a recently developed OBIKA^19^ and uses whole proteome immobilized on *N*-hydroxy-succinimide (NHS)-activated Sepharose beads under native conditions as input for *in vitro* reactions (Figure 1A). Upon cellular lysis, endogenous serine-threonine protein phosphatases are effectively inhibited through the addition of the nonselective phosphoprotein phosphatase (PPP) inhibitor microcystin-LR (MCLR), which binds covalently to the active sites of catalytic PPP subunits (Figure 1A).^20^ This leads to an increase in both the number and intensity of phosphorylation events that can be detected by liquid chromatography-tandem MS (LC-MS/MS)-based phosphoproteomics (Figure 1B). As the cellular lysis is performed under native conditions (see STAR Methods for details), protein complexes remain intact and kinases remain active. Thus, the addition of ATP supports *in vitro* kinase reactions of endogenous kinases, which further increase the numbers and intensities of phosphosites (Figure 1B). Finally, the simultaneous inclusion of both ATP and MCLR leads to a maximal increase in detected phosphosites, i.e., approximately 35,000 in our setting (Figure 1B).

Following *in vitro* kinase reactions, the proteome is coupled to NHS-activated beads to perform *in vitro* dephosphorylation reactions by the addition of a purified phosphatase of interest (Figure 1A). As a positive control, we added purified λ-phosphatase (Figure 1C), a broadly acting enzyme capable of dephosphorylating phosphoserine, -threonine, and -tyrosine residues. Compared to control samples to which no phosphatase was added, the addition of λ-phosphatase led to the dephosphorylation of a total of 25,867 phosphosites (Figure 1C; *n* = 3, false discovery rate [FDR] <0.05, t test; Table S1). This outcome serves as evidence of the efficacy of our workflow.

### OBIPhAs of PP1 and PP2A complexes highlight broad phosphatase reactivities

**Figure 2:**
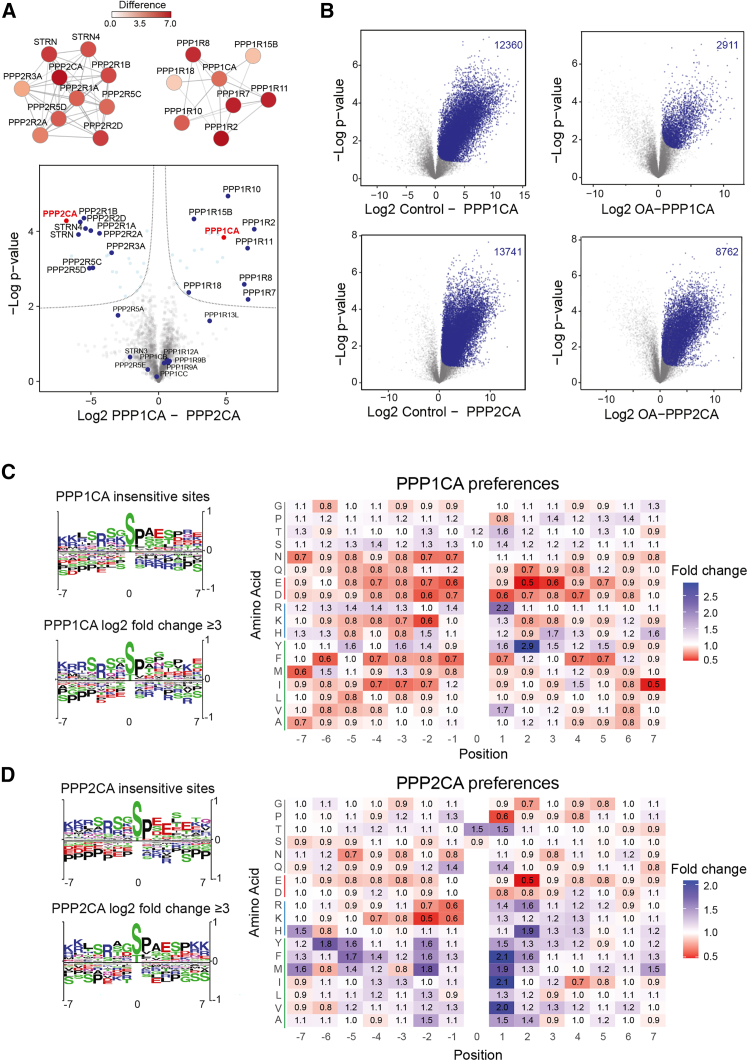
Global phosphoproteome-wide *in vitro* phosphatase assays of PP1 and PP2A (A) Volcano plot highlighting identified phosphatase subunits in StrepHA-PPP1CA (right) and StrepHA-PPP2CA (left) purifications (marked in red) under native conditions. Dotted lines mark significance threshold; significantly enriched proteins marked in blue (*n* = 3 biological replicates, FDR <0.05). Significantly enriched phosphatase subunits are annotated and highlighted as protein-interaction networks using information present in STRING DB.^25^ (B) Differentially regulated PPP1CA/PPP2CA sites, compared to either untreated control sample (left), or phosphatase- and okadaic acid-treated samples (OA, right). *In vitro* significantly regulated sites are highlighted in blue (*n* = 3 biological replicates; FDR <0.05). (C and D) Motif and preference analyses of PPP1CA- and PPP2CA-sensitive sites. In motif analyses phosphatase-sensitive and OA-sensitive and insensitive sites are compared. Coloring corresponds to biochemical properties of residues. In preference analyses, the relative abundance of a given amino acid in a given position is indicated comparing phosphatase- and OA-sensitive (fold change of ≥3) to phosphatase-insensitive phosphosites. Colored lines next to amino acid names signify amino acid characteristics: green for hydrophobic, blue for basic, red for acidic, and gray for neutral amino acids. See also Table S2.

Following the successful test of our workflow, we proceeded to evaluate the method’s applicability by examining two prominent serine/threonine protein phosphatases, PP1 and PP2A. Both PP1 and PP2A function as holoenzymes, comprising catalytic subunits (PPP1CA-C and PPP2CA-B, respectively) and a diverse array of scaffolding and/or regulatory subunits.^21^^,^^22^ The purification of these phosphatases was done with a primary emphasis on preserving the integrity and complexity of their respective holocomplexes, not discriminating between different variants. Briefly, HeLa cells expressing StrepHA-tagged variants of PPP1CA and PPP2CA were used to purify complexes using a mild detergent containing buffer (see STAR Methods for details). To check the purity and reproducibility of our preparations, we conducted quantitative MS analyses. In both cases, we reproducibly and significantly enriched various PP1 and PP2A-specific regulatory subunits in the preparation of the respective catalytic subunit (Figure 2A). As we worked under native conditions, PP1 and PP2A complexes were not pure but enriched next to many other proteins (Table S2A). Complexome profiling based on blue native (BN)-PAGE-MS^23^ and western blots highlight that several macromolecular PP1 and PP2A complexes were enriched in each of the respective preparations (Figures S1A and S1B), indicating that we managed to (1) preserve holoenzymes and (2) enrich specific phosphatase complexes. We identified a minor fraction of PPP1CA in PPP2CA preparations and vice versa, which implies that the catalytic phosphatase subunits themselves interact, as has been suggested.^24^ However, in both cases the copurifying catalytic subunit was more than 25 times less abundant than the target subunit (Figure 2A; Table S2A).We therefore conclude that preparations can be used to study PP1- and PP2A-specific phosphatase-substrate interactions *in vitro*, with the constraint that not a single PP1 or PP2A holocomplex is enriched, but a mixture of respective complexes, and that copurifying contaminants must be taken into account.

We analyzed activities of purified phosphatase complexes by the p-nitrophenyl phosphate (pNPP) assay (Figures S1C and S1D). Both PPP1CA and PPP2CA exhibited *in vitro* activity in kinetic experiments, also highlighting the presence of nonspecific, MCLR-insensitive phosphatase activity. PP1 was more active than PP2A and responded differentially to the addition of the inhibitor okadaic acid (OA), 2 nM OA having a similar effect on PPP2CA as 2 μM OA on PPP1CA, corroborating published data.^26^^,^^27^ Upon confirmation of enzymatic activity of the purified phosphatase complexes and their differential susceptibility to inhibition by OA, we engaged in a comprehensive *in vitro* phosphoproteomic investigation utilizing both mixtures of purified PP1 and PP2A complexes, with and without OA supplementation. A control sample without phosphatase addition served as baseline, representing the experimentally detectable phosphoproteome. Target sites had to be dephosphorylated upon addition of phosphatase complexes, and dephosphorylation had to be blocked upon the addition of OA, to discriminate between potentially specific from nonspecific, inhibitor-insensitive events. We performed OBIPhAs, followed by quantitative MS-based phosphoproteomics employing data-independent acquisition (DIA). Our analyses revealed a discernible landscape of phosphatase-sensitive phosphorylation sites, yielding a total count of 12,360 PP1- and 13,741 PP2A-sensitive sites (Figure 2B; *n* = 3, FDR <0.05, t test; Tables S2B and S2C). Addition of OA preserved 63.8% of PP2A-sensitive sites (8,762 of the initial 13,741 sites), whereas only 23.6% of PP1-sensitive sites were protected (2,911 of 12,360 sites). This differential response also supports our interpretation that PPP1CA contaminations in PPP2CA preparations are negligible. Finally, we compared phosphosites surrounding amino acid sequences of PP1 and PP2A OA-sensitive to respective OA-insensitive sites by generating sequence motifs and analyzing phosphatase preferences independent of the amino acid occurrence in a certain position (Figures 2C and 2D). In agreement with published data,^28^ we detected no strong trends. In both cases, acidic residues C-terminally of the phosphosite seem to be deselected,^14^ which is in contrast to PP6.^29^ In both cases, the arginine in the −3 position also seems to not be specific but rather a characteristic of the purified phosphopeptides, potentially reflecting the large number of kinases with preferences of basophilic residues N-terminally to the phosphorylation site.^30^ This discrepancy with published PP1 data might be linked to the fact that we did not use recombinant PPP1CA but rather a mixture of different holoenzymes.^28^ The preference analysis additionally indicates that basic residues are favored over acidic ones—N-terminal in the case of PP1 and C-terminal in the case of PP2A—and that phosphorylated threonine is preferred compared to phosphorylated serine.^14^^,^^28^ PP1 seems to prefer a tyrosine in +2, whereas PP2A prefers hydrophobic residues in +1. However, as most sites are SP sites with proline in +1, this result must be treated with caution (Figures 2C and 2D). Taken together, both motif and preference analyses indicate a limited influence of the phosphosites’ surrounding amino acid residues on phosphatase specificity, except for a negative influence of acidic amino acid residues.

### Combination of *in vitro* and *in vivo* phosphoproteomic assays characterizes bona fide PP2A target sites

**Figure 3:**
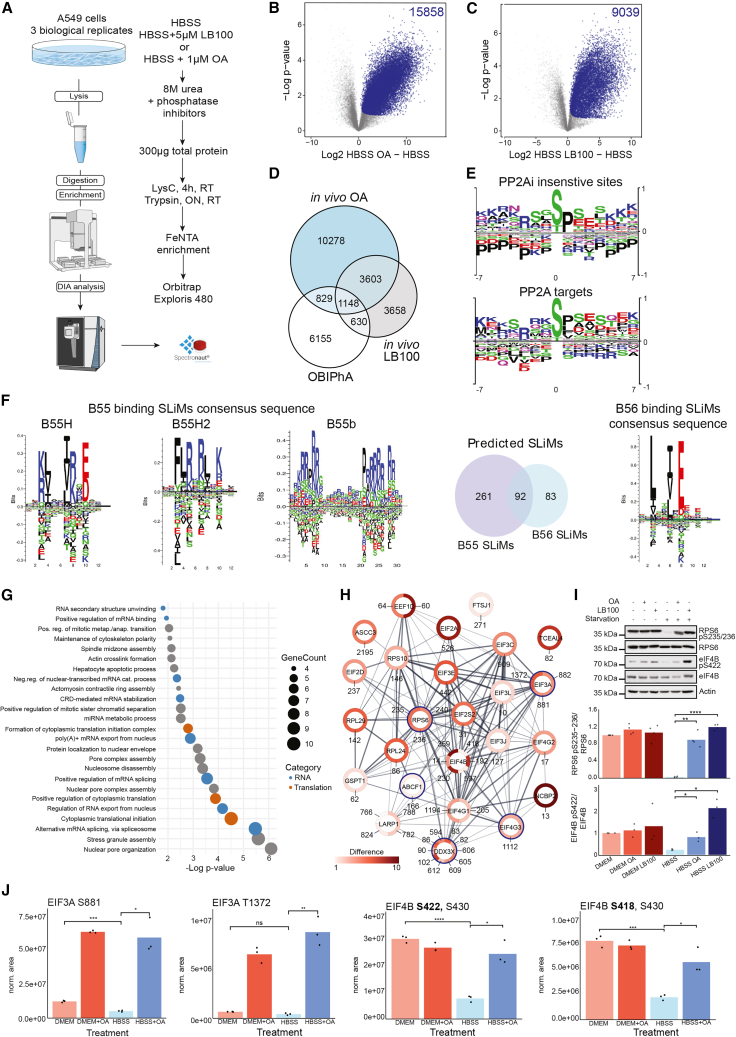
Characterization of bona fide PP2A target sites (A) Label-free quantitative phosphoproteomic workflow. A549 cells were starved for 2 h in Hank’s balanced salt solution (HBSS) or starved and treated with a PP2A inhibitor (LB100 or OA). (B and C) Significantly regulated phosphosites for PP2A comparing starvation with and without PP2A inhibition (*n* = 3 biological replicates). Significant sites are highlighted in blue (FDR <0.05). (D) Comparison of OBIPhA and *in vivo* analyses. Venn diagram highlights the overlap of identified phosphosites using the different experimental approaches. Bona fide PP2A target sites are defined as being regulated *in vivo* by both OA and LB100 treatment, and *in vitro* (i.e., 1,148 sites). (E) Motif analyses of *in vivo* insensitive vs. identified bona fide PP2A target sites. (F) Consensus sequences and the numbers of the identified B55 (B55H, B55H2, and B55b) and B56 SLiMs in the bona fide PP2A target sites. (G) GO enrichment analysis using STRING DB highlighting biological processes of PP2A target proteins. (H) Protein interaction network highlighting known interactions of PP2A target proteins that are involved in “cytoplasmic protein translation.” Network nodes represent proteins, network edges represent interactions, thickness of edges represent the confidence of interactions (STRING DB). Color scale of circles indicates magnitude of significant regulation of target sites as identified by *in vivo* proteomics. Blue circles indicate proteins with SLiM motifs. (I) Phosphosite-specific western blots corroborating that Ser235 and Ser236 of RPS6 and Ser422 of eIF4B are bona fide PP2A target sites as identified by proteomic approaches. Note: eIFB was identified as a target protein by proteomics, but the site Ser422 did not pass the significance cutoff. Shown is one out of three biological replicates. Bar plot shows quantification of three or four replicates relative to the loading control β-actin (black dots). ∗∗∗*p* < 0.001; t test. (J) Targeted, phosphosite-specific mass spectrometry (PRM) for eIF3A Ser881 and Ser1372 as well as eIF4B Ser418 and Ser422. Bar plot shows quantification of three replicates (black dots). ∗*p* < 0.05; ∗∗∗*p* < 0.001; t test. Note: eIF4B peptides carry two phosphosites—the highlighted one in bold font and Ser430. As the latter was also detected in a singly phosphorylated variant not being regulated (Figure S2E), the depicted abundance changes should reflect the regulation of the indicated sites. See also Table S3.

We assume that our *in vitro* whole-proteome phosphatase assay under native conditions is more specific than phosphatase assays on the peptide level or assays using single recombinant proteins, due to the competition of native substrates. Nevertheless, we cannot address the number of non-physiological PP2A target sites by *in vitro* analyses only. To generate a list of bona fide (i.e., physiologically observed) phosphatase target sites, we concentrated on PP2A and performed *in vivo* phosphoproteomics (Figure 3A). Briefly, we generated three biological replicates of three conditions: A549 cells in (1) starvation conditions, which leads to increased phosphatase activity compared to growth conditions as indicated by pNPP hydrolysis,^19^ (2) starvation conditions in the presence of the PPP inhibitor OA,^31^ and (3) starvation conditions in the presence of the PP2A and PP5 inhibitor LB100.^32^^,^^33^ PP2A target sites should be dephosphorylated in condition (1) and phosphorylated in conditions (2) and (3). The usage of two different inhibitors that both target PP2A as common phosphatase should support the discrimination of PP2A sites from other PPP sites, which are only targeted by one of the inhibitors or are less sensitive to OA,^31^ respectively. Cells were lysed and processed as depicted in Figure 3A. Discovery-driven DIA-based phosphoproteomics led to the quantification of a total of 41,932 phosphorylation events, with a localization probability above 75%. Of these, 26,338 could be normalized to their source proteins for OA treatment and 17,261 sites for LB100. OA-sensitive (15,858 sites) and LB100-sensitive (9,039 sites) sites overlapped by 4,751 sites, which represent significantly *in vivo*-regulated potential PP2A target sites according to the experimental design (Figures 3B–3D; FDR <0.05, t test; Tables S3A and S3B).

Next, these sites were compared to our *in vitro* phosphoproteome to identify bona fide PP2A target sites (i.e., sites that are significantly dephosphorylated by PP2A complexes both *in vitro* and *in vivo*) (Figure 3D; Table S3C). This led to the identification of 1,148 PP2A target sites on 586 source proteins. Compared with published *in vivo* PP2A-dependent phosphoproteomes, we share 384 target proteins and 240 target sites (Figure S2A).^18^^,^^34^ This rather small overlap is likely because in the respective *in vivo* studies indirect and direct effects of PP2A inhibition were not discriminated, as the overlap is tripled by considering only our *in vivo* data (Figure S2B). The increased noise in peptide-based phosphosite localization likely leads to a smaller overlap in phosphosites compared to target proteins. Compared to published data, *in vitro* we detect more multi-dephosphorylated proteins, which may be a protein abundance bias of the *in vitro* assay and MS detection; however, this is lost by filtering the *in vitro* with the *in vivo* data (Figure S2C).

Phosphatase motif analysis again did not lead to the identification of clearly preferred amino acids surrounding the PP2A target sites (Figure 3E). As PP2A target recognition is partially guided by SLiMs, we scanned our list of 1,148 target sites for the presence of SLiMs that are recognized by the 2 regulatory subunit families B55 (PPP2R2) and B56 (PPP2R5), as these were the main complexes that we purified (Figure 2A, *PPP2CA-PPP2R1A/B-PPP2R2A/D* and *PPP2CA-PPP2R1A/B-PPP2R5C/D*). Three B55 SLiMs were used: in addition to B55b,^35^ the recently discovered B55H and B55H2 were used in the search (Figure 3F).^36^^,^^37^ To ca. 30% of target sites potential SLiMs could be assigned, the majority of which are in disordered regions. 261 sites are located on target proteins that carry B55 SLiMs, 83 sites are located on target proteins that carry B56 SLiMs, and 92 sites are located on proteins that carry both SLiMs (Figure 3F; Table S3C). A significant fraction of B56 SLiMs have larger spacers between them and target phosphorylation sites than B55 spacers. The latter exhibits a Gaussian distribution, with shorter spacers at higher abundance (Figure S2D).

The longer spacers for B56 SLiMs might be derived from the PP2A-B56δ holoenzyme. B56δ is encoded by *PPP2R5D*, and its enrichment is slightly higher than that of *PPP2R5C* (Figure 2A). *PPP2R5C* encodes B56γ1, which is the shortest form in the B56 family that is not known to be regulated. The B56δ holoenzyme, on the other hand, is highly regulated. B56δ harbors long intrinsically disordered regions at both N and C termini that form cross-arm interactions against the holoenzyme core suppressing the SLiM-binding pocket, the active site, and the path between them, resulting in reduced basal SLiM binding.^38^ It is likely that the direct path from the SLiM-binding site to the phosphatase active site is not cleared upon holoenzyme activation, and that the N/C arms might remain associated upon the activation of the holoenzyme. The B56δ substrates need longer spacers for their phosphorylation site to reach the phosphatase active site.

Next, we analyzed the potential physiological relevance of identified PP2A target sites by performing Gene Ontology (GO) enrichment of respective target proteins. These were involved in numerous processes ranging from “nuclear pore organization” to the “formation of cytoplasmic translation initiation complex” (Figure 3G), corroborating published data.^35^^,^^39^ Due to the known crosstalk between mammalian target of rapamycin complex (mTORC1) and PP2A signaling and its relevance for protein translation and autophagy,^19^^,^^40^ we focused on targets that could be linked to protein translation. Indeed, we could generate a protein-protein interaction network of 25 PP2A target proteins that play a role in protein translation (Figure 3H), supporting the conclusion that one function of PP2A is the regulation of protein translation.^41^ Five of these proteins, RPS6, EIF3A, ABCF1, DDX3X, and EIF4G3, also carried B55 SLiMs. PP2A-dependent alteration of RPS6 phosphorylation on sites Ser235 and Ser236, which were recently shown to be modulated *in vivo* in Jordan’s syndrome,^39^ and eukaryotic initiation factor 4B (eIF4B) phosphorylation on site Ser422 could be validated by western blot using site-specific antibodies (Figure 3I). In addition, we performed targeted MS experiments based on parallel reaction monitoring (PRM) to study phosphorylation events on Ser881 and Thr1372 of eIF3A and Ser422 and Ser418 of eIF4B, sites for which no phosphosite-specific antibodies were available. As in the case of site-specific western blots, PRM assays agreed with our phosphoproteomic data—starvation leading to a decreased phosphorylation and OA treatment interfering with this decrease—supporting the interpretation that these sites are indeed bona fide PP2A target sites (Figures 3J and S2E).

### Characterization of the PP2A-PPP2R5E/B56ε target repertoire

**Figure 4:**
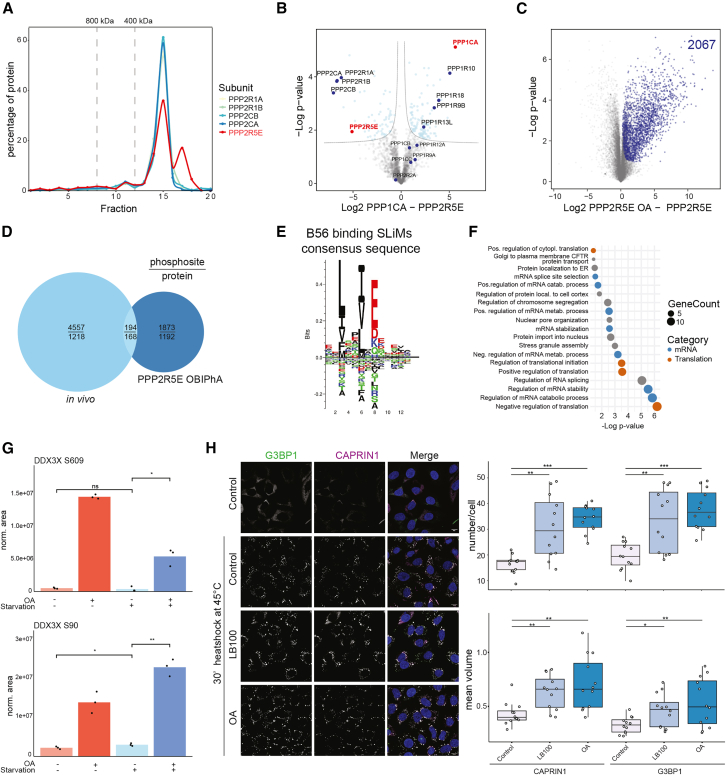
Characterization of bona fide PP2A-PPP2R5E target sites (A) Complexome profiling of StrepHA-PPP2R5E purifications indicates enrichment of holocomplexes. (B) Volcano plot highlighting identified phosphatase subunits in StrepHA- PPP2R5E (left) and StrepHA-PPP1CA (right) purifications (marked in red) under native conditions. Dotted lines mark significance threshold, significantly enriched proteins marked in blue (*n* = 3 biological replicates, FDR <0.05). Significantly enriched phosphatase subunits are annotated. (C) Significantly regulated phosphosites for PPP2R5E-PP2A complexes being OA sensitive (*n* = 3 biological replicates). Significant sites are highlighted in blue (FDR <0.05). (D) Comparison of OBIPhA and *in vivo* analyses. Venn diagram highlights the overlap of identified phosphosites using the different experimental approaches. Bona fide PPP2R5E-PP2A target sites are defined as being regulated *in vivo* and *in vitro* (i.e., 194 sites on 168 proteins). (E) SLiMs in the bona fide PPP2R5E/B56ε-PP2A targets. (F) GO enrichment analysis using STRING DB highlighting biological processes of PPP2R5E-PP2A target proteins. (G) Targeted, phosphosite-specific MS analysis (PRM) for DDX3X Ser90 and Ser609. Shown is the quantification of three replicates (black dots). ∗*p* < 0.05; ∗∗*p* < 0.01; t test. (H) IF of CAPRIN1 and G3BP1. Shown are exemplary images of *n* = 3 biological replicates. Nuclei are stained in blue. Scale bar, 10 μm. Box plots show quantifications of 12 images of *n* = 3 biological replicates, boxes are drawn according to ggplot2 standard settings. White dots indicate average stress granule number/mean volume per cell and image. ∗*p* < 0.05; ∗∗*p* < 0.01; t test. See also Table S4.

To highlight the usability of our approach for identifying complex-specific PP2A target sites, we focused on PPP2R5E/B56ε-containing complexes. PPP2R5E has been linked to apoptosis and cancer^42^^,^^43^; however, its influence on the PP2A target repertoire is not well understood. In our cell model, B56ε-containing complexes are low abundant, and hence underrepresented in the PPP2CA purifications (Figure 2A; Table S2A). Using a StrepHA-tagged variant, we purified B56ε-containing complexes under native conditions (Figures 4A and 4B; Table S4A). Interestingly, B56ε seems to interact to a similar extent with both catalytic subunits, PPP2CA and PPP2CB, as well as with both scaffold subunits, PPP2R1A and PPP2R1B. Using respective complexes in OBIPhAs led to the identification of 2,067 OA-sensitive target sites (Figure 4C; Table S4B); of these, 194 sites on 168 proteins were also identified *in vivo* (Figure 4D; Table S4C), characterizing them as bona fide PP2A-B56ε-target sites. A handful of B56 SLiMs were found for those B56ε-targeting substrates (Figures 4E and S3A).^8^^,^^44^^,^^45^^,^^46^ Intriguingly, the spacers for B56ε SLiMs are dominantly long (Figure S3A). Although there is no structural information on this holoenzyme, it is likely to be highly regulated as B56δ also has long disordered arms.

Respective target proteins were associated with diverse processes ranging from sister chromatid segregation to cytoskeletal organization, two processes that have been linked to B56ε (Figure 4F).^47^^,^^48^ Reassuringly, proteins involved in the regulation of translation also carried PP2A-B56ε target sites, specifically proteins that are linked to stress granule assembly, like the DEAD-box helicase DDX3X.^49^ Due to the lack of site-specific antibodies, we again performed PRM analyses and could show that—in agreement with our phosphoproteomic data—Ser90 and Ser609 fulfill all the criteria of being PP2A target sites; these are, however, not strongly regulated by starvation (Figure 4G). Stress granules are cytosolic deposits containing translation factors and translationally repressed mRNAs, and are used to control protein synthesis in a variety of stress conditions.^50^ The cytosolic sequestration of translation factors is linked to a global inhibition of protein synthesis, one conserved mechanism being the phosphorylation of the eukaryotic translation initiation factor eIF2α by the kinase GCN2.^51^^,^^52^ Reactivation of translation is dependent on the dephosphorylation of eIF2α by PP1,^53^ increased PP1 activity being linked to stress granule disassembly.^54^ As a role of PP2A in stress granule assembly has not been described, we monitored heat stress-dependent stress granule assembly by IF using the two marker proteins CAPRIN1 and G3BP1.^55^^,^^56^ Whereas treatment with both LB100 or OA had no influence on stress granule assembly in control conditions (Figure S3B), it led to an increase in number and size of stress granules in heat stress, indicating that similar to PP1, PP2A activity is also negatively linked to stress granule assembly (Figure 4H).

In conclusion, we developed an OBIPhA that supports phosphoproteomic screening approaches. Performing *in vivo* and *in vitro* assays on proteome scale will support the streamlined characterization of phosphatase targets. Whereas we used the catalytic subunits of PP1 and PP2A for protein complex purifications isolating a rather crude and complex mixture of different holocomplexes, tagging and purification via regulatory subunits will support the characterization of specific holocomplex target repertoires, as exemplified by the B56ε-containing PP2A complex. Coupling of *in vitro* (de)phosphorylation reactions to bead-immobilized native protein complexes supports automation of workflows, MS sample preparation, and analysis. In principle, any enzyme-based *in vitro* reaction can be coupled with proteome input if covalent enzyme inhibitors exist that can be used to block endogenous enzymes that are still active under native conditions. We used this approach successfully for kinases and phosphatases and currently elaborate its suitability for *in vitro* ubiquitination reactions.

### Limitations of the study

The outlined workflow critically depends on the availability of covalent enzyme inhibitors. In our case, we used MCLR to permanently block all endogenous PPP-type phosphatases. Hence, we can only study target sites of PPP-type phosphatases and cannot comment on potential effects on, for example, tyrosine or dual-specificity phosphatases. As we work under native conditions, these phosphatases are still active, and it can be envisioned that exogenously added PP1 or PP2A might affect activities of respective endogenous phosphatases. The usage of additional phosphatase-specific inhibitors like OA and LB100 in *in vitro* experiments are important controls, as we used rather crude enrichments also exhibiting MCLR non-sensitive phosphatase activity; however, crosstalk with non-PPP-type phosphatases cannot be excluded. Additional catalytic subunits of phosphatases might copurify with the phosphatase of interest, which might explain the limited effect of OA treatment in our experiments, blocking only 63.4% of potential PP2A dephosphorylations. Therefore, a differential analysis that considers only OA-sensitive sites is important. As an alternative, catalytically inactive variants of phosphatases of interest could be used, or SLiM inhibitor sequences could be expressed to specifically study single phosphorylation events. The combination of *in vivo* and *in vitro* phosphoproteomic data is vital to identify physiologically relevant phosphosites. As *in vitro* experiments are performed in detergent-containing buffers, cellular membranes are disrupted and spatial constraints of dephosphorylation reactions are lost. 28.6% of our bona fide PP2A targets were also regulated by PP1 *in vitro*. Whether these sites are indeed regulated also *in vivo* by both phosphatases will have to be addressed in future studies. Finally, we described a role of PP2A-PPP2R5E/B56ε-containing complexes in stress granule disassembly and identified several bona fide target sites on stress granule-associated proteins. Whether these sites are relevant for stress granule turnover or the control of protein translation will have to be determined in future studies.

## Resource availability

### Lead contact

Further information and requests for resources and reagents should be directed to and will be fulfilled by the lead contact, Jörn Dengjel (joern.dengjel@unifr.ch).

### Materials availability

Generated materials are freely available via the lead contact.

### Data and code availability

• Discovery proteomics data are freely available via the PRIDE repository (https://www.ebi.ac.uk/pride/), data identifier PXD050485 and PXD060829. Targeted proteomics data are freely available from Panorama (https://panoramaweb.org/4DfEpv.url). Unprocessed raw western blots and imaging data are available via Mendeley Data, V1 (https://doi.org/10.17632/h9w2yv39xp.1).
• This paper does not report original code.
• Any additional information required to reanalyze the data reported in this paper is available from the lead contact upon request.

## Acknowledgments

This work was supported by the Canton and the University of Fribourg as part of the SKINTEGRITY.CH collaborative research project, the 10.13039/501100001711Swiss National Science Foundation (grant nos. 310030_212187 and 310030_184781 to J.D.), the 10.13039/501100004784Novartis Foundation for Medical-Biological Research (grant no. 21C173 to J.D.), and by R01 funding from the National Institute of Health (grant no. GM 145811 to Y.X.). We thank the University of Wisconsin Carbone Cancer Center Cancer Informatics Shared Resource, supported by P30 CA014520, for use of its services, Michael Stumpe and Alexandre Leytens for MS support, Irene M. Ong for support with the Labkey interface, and the Bioimage Core Facility for imaging support.

## Author contributions

Conceptualization, Z.H. and J.D.; methodology, M.B., Z.H., H.E., G.L., C.R., C.V., D.S.S., S.J.M., and S.K.-P.; formal analysis, M.B., Z.H., H.E., C.V., S.K.-P., Y.X., and J.D.; writing – original draft, M.B., Y.X., and J.D.; writing – review & editing, M.B., Z.H., H.E., Y.X., and J.D.; visualization, M.B., Z.H., H.E., Y.X., and J.D.; funding acquisition, Y.X. and J.D.

## Declaration of interests

The authors declare no competing interests.

## STAR★Methods

### Key resources table

**Table:** 

REAGENT or RESOURCE	SOURCE	IDENTIFIER
**Antibodies**		

Rabbit polyclonal anti RPS6-pS235/236	ProteinTech	Cat#29223-1-AP; RRID:AB_2918253
Mouse monoclonal anti RPS6	Santa Cruz Biotech	Cat#sc-74459; RRID:AB_1129205
Mouse monoclonal anti β-Actin	Santa Cruz Biotech	Cat#sc-47778HRP; RRID:AB_2714189
Mouse monoclonal anti PP2A-B55-a	Santa Cruz Biotech	Cat# sc-81606; RRID:AB_2252935
Rabbit polyclonal anti PP2A C subunit	Cell Signaling	Cat# 2038; RRID:AB_2169495
Mouse monoclonal anti PPP2R1A/B	Santa Cruz Biotech	Cat# sc-13600; RRID:AB_628178
Mouse monoclonal anti PPP2R5E	Santa Cruz Biotech	Cat# sc-376176; RRID:AB_10986266
Mouse monoclonal anti MYPT1	Santa Cruz Biotech	Cat# sc-514261
Rabbit polyclonal anti EIF4B	Cell Signaling	Cat# 3592; RRID:AB_2293388
Rabbit polyclonal anti EIF4B pS422	Cell Signaling	Cat# 3591; RRID:AB_2097522
Mouse monoclonal anti PPP1CA	Santa Cruz Biotech	Cat# sc-7482; RRID:AB_628177
Rabbit polyclonal anti Caprin1	ProteinTech	Cat#15112-1-AP RRID:AB_2070016
Mouse monoclonal anti G3BP1	Santa Cruz Biotech	Cat#sc-365338 RRID:AB_10846950
Donkey anti-mouse IgG (H + L) Secondary, Alexa Fluor 488 Conjugated	Thermo Scientific	Cat#A21202 RRID:AB_141607
Goat anti-rabbit IgG (H + L), Alexa Fluor 633 Conjugated	Thermo Scientific	Cat#A21071 RRID:AB_141419
Peroxidase-conjugated goat anti-rabbit IgG	Jackson Immuno Research	Cat#111-035-045; RRID:AB_2337938
Peroxidase-conjugated goat anti-mouse IgG	Jackson Immuno Research	Cat#115-035-062; RRID:AB_2338504

**Bacterial and virus strains**		

E. coli DH5a	CGSC	Cat#12384

**Chemicals, peptides, and recombinant proteins**		

Dulbecco’s Modified Eagle Medium (DMEM)	PAN Biotech	Cat#P04-04510
Fetal Bovine Serum (FBS)	BioWest	Cat#S181B-500
Trypsin for cell culture	PAN Biotech	Cat#P10-023100
Penicillin-Streptomycin	PAN Biotech	Cat#P06-07100
PP2A inhibitor LB100	RayBiotech	Cat#332-11915-2
PP2A inhibitor okadaic Acid	Merck	Cat#O9381
Hank’s Balanced Salt Solution (HBSS)	Gibco	Cat#14025-100
Dulbecco’s Phosphate Buffered Saline (DPBS)	PAN Biotech	Cat#P04-36500
Strep-Tactin XT-4Flow Slurry	IBA	Cat#2-5030-010
Microcystin LR	Enzo Life Sciences	Cat#ALX-350-012-M001
Doxycycline	Sigma-Aldrich	Cat#D9891
Nitrocellulose membrane 0.45 mm	Amersham	Cat#GE10600000
Benzonase	Sigma-Aldrich	Cat#1.01697.0001
PhosSTOP	Roche	Cat#04-906-837-001
Proteases Inhibitor Cocktail	Roche	Cat#11-697-498-001
NHS-activated Sepharose 4 fast flow	GE Healthcare	Cat#17-0906-01
Trifluoroacetic acid (TFA)	Sigma-Aldrich	Cat#302031
Formic acid (FA)	Merck	Cat#5.43804.0250
Ammonia solution 25%	Merck	Cat#5438300250
Trypsin	Promega	Cat#V5113
ATP	Sigma-Aldrich	Cat#A6419
Vivacon 500, 100′000 MWCO	Sartorius	Cat#VN01H42
Lys-C	FUJIFILM Wako Pure Chemical Corporation	Cat#129-02541
HR-X Column	Macherey-Nagel	Cat#730936P45
MS-grade Water	VWR	Cat#23595.328
MS-grade Acetonitrile	VWR	Cat#20060.32
Biotin	Sigma-Aldrich	Cat#B4501
Collagen I	ThermoFisher	Cat#A10483-01
Hoechst 3342	Sigma-Aldrich	Cat#14533
ProLong Gold antifade	ThermoFisher	Cat#P36931
Horse serum	ThermoFisher	Cat#16050

**Critical commercial assays**		

Pierce BCA Protein Assay Kit		Cat#PIER23225
WesternBright ECL	Advansta	Cat#K-12045-D50
SuperSignal West Femto Chemiluminescent Substrate	Pierce	Cat#PIER34096

**Deposited data**		

MS raw data	ProteomeXchange	PXD050485; PXD060829
Western blot uncropped images	Mendeley Data	https://doi.org/10.17632/h9w2yv39xp.1
PRM data	PanoramaWeb	https://panoramaweb.org/4DfEpv.url

**Experimental models: Cell lines**		

A549 cells	ATCC	Cat#CCL-185
HeLa cells	ATCC	Cat#CCL-2
SHA-PPP1CA HeLa (Figures 2 and S2)	This study	N/A
SHA-PPP2CA HeLa (Figures 2, 3, and S2)	This study	N/A
SHA-PPP2R5E HeLa (Figures 4 and S3)	This study	N/A

**Oligonucleotides**		

oNDS286	This study	5′ cgatgaaaatttatattttcaag gtatggacgagaaggtgttcacc 3′
oNDS287	This study	5′ catatccagtcactatggt cgacctgcagttacaggaagta gtctggggtacg 3′
oNDS292	This study	5′ cgatgaaaatttatatttt caaggtatgtccgacag cgagaagc 3′
oNDS293	This study	5′ catatccagtcactatggtcga cctgcagttatttcttggctttgg cggaattgc 3′

**Recombinant DNA**		

psPAX2	Addgene	Plasmid #12260
pMD2.G	Addgene	Plasmid #12259
pBabe Zeo PPP2CA WT	Addgene	Plasmid #10689
pSKP-173 (StrepHA-PPP2CA)	Generated in this study	N/A
pSKP-176 (StrepHA-PPP1CA)	Generated in this study	N/A
pSKP-204 (StrepHA-PPP2R5E)	Generated in this study	N/A

**Software and algorithms**		

Spectronaut	Biognosys	Version 17 or 19
Perseus	Tyanova et al.^57^	https://maxquant.net/perseus/
Cytoscape	Shannon et al.^58^	https://cytoscape.org/
ClueGo	Bindea et al.^59^	https://apps.cytoscape.org/apps/cluego
PhosphoSitePlus	PhosphoSitePlus, NIH	https://www.phosphosite.org/staticMotifAnalysis.action
Seq2Logo	Thomsen et al.^60^	https://services.healthtech.dtu.dk/services/Seq2Logo-2.0/
R	https://www.r-project.org/	N/A
ImageJ	National Institutes of Health	https://imagej.nih.gov/ij/index.html
Skyline	MacLean et al.^61^	https://skyline.gs.washington.edu/project/home/software/Skyline/begin.view
Imaris v10.2.0	Bitplane, Oxford Instruments	https://imaris.oxinst.com/

### Experimental model and study participant details

#### Cell culture and treatments

A549 and HeLa cells (CCl-185 and CCL-2 respectively, authenticated by genotyping (Microsynth) and tested negative for mycoplasma) were cultured in Dulbecco’s Modified Eagle Medium (DMEM, PAN Biotech, P04-04510) supplemented with 10% fetal bovine serum (FBS, BioWest, S181B-500) and 100 units/ml penicillin and 0.1 mg/mL streptomycin (PAN Biotech, P06-07100) at 37°C and 5% CO_2_. For treatments, cells were seeded to reach 80% confluency and treated 24 h after for 2 h prior to harvesting with 5 μM PP2A inhibitor LB100 (RayBiotech, 332-11915-2) or 1 μM okadaic acid (Merck, O9381). Starvation with Hank’s balanced salt solution (HBSS, Gibco 14025-100) was performed after washing cells twice with Dulbecco’s phosphate buffer saline (DPBS, PAN Biotech, P04-36500). For collection, all dishes were washed twice with ice-cold PBS (PAN Biotech X0515-500), scraped, and collected in tubes followed by centrifugation to pellet the cells. The supernatant was discarded, and pellets were stored at −80°C.

#### Lentiviral vectors

The following constructs were obtained from Addgene: psPAX2 (Plasmid #12260), pMD2.G (Plasmid #12259), pBabe Zeo PPP2CA WT (Plasmid # 10689). Inducible lentiviral vectors for exogenous expression of StrepHA-PPP2CA (pSKP-173), StrepHA-PPP1CA (pSKP-176) and StrepHA-PPP2R5E (pSKP-204 recombinant proteins were obtained as follows. The PPP2CA ORF was amplified by PCR using pBabe Zeo PPP2CA WT vector as template and oNDS286 (5′ cgatgaaaatttatattttcaaggtatggacgagaaggtgttcacc 3′) and oNDS287 (5′ catatccagtcactatggtcgacctgcagttacaggaagtagtctggggtacg 3′) as primers, whereas the PPP1CA and PPP2R5E ORFs were amplified by PCR from a first-strand synthesis cDNA generated from HEK293tRNA, using the oNDS292 (5′ cgatgaaaatttatattttcaaggtatgtccgacagcgagaagc 3′) and oNDS293 (5′ catatccagtcactatggtcgacctgcagttatttcttggctttggcggaattgc 3′) primers for PPP1CA, and oGL5 (5′ggggacaagtttgtacaaaaaagcaggctgccaccatgtacccatacgatgttcctgactatgccatgtcctcagcaccaactactc 3′) and oGL6 (5′ ggggaccactttgtacaagaaagctgggtttaagttggaattattccatcacg 3′) for PPP2R5E. For PPP2R5E the cDNA was first cloned into pDONR201 using gateway BP recombination (Thermofisher, 11789020) following manufacturer’s recommendations, to produce pSKP-202, which was used as template for further cloning into lentiviral vectors. PPP2R5E was then PCR amplified from pSKP-202 using oGL7 (5′ cgatgaaaatttatattttcaaggtatgtcctcagcaccaactactc 3′) and oGL8 (5′ catatccagtcactatggtcgacctgcagttaagttggaattattccatcacg 3′) primers. The StrepHA tag was amplified by PCR using primers oNDS107 (5′ accttgaaaatataaattttcatcggccgctttttcgaactgc 3′) and oNDS111(5′cagatcgcctggagaattggctagcgaattcgccaccatgtacccatacgatgttcctgactatgccg3′) primers, and combinations of StrepHA and PPP2CA or PPP1CA or PPP2R5E ORF PCR products were cloned into pSKP-32, previously digested between NheI and PstI sites, via Gibson assembly (New England Biolabs, E2611S), following manufacturer’s instructions. All resulting plasmids were sequence verified before processing with lentiviral particle production. pSKP-173 and pSKP-176 constructs contain 2x Strep-tactin tags before their respective ORF, whereas pSKP-204 contains only 1.

#### Lentiviruses: Transfection into packaging cells and target cell infection

Viral particles containing the pSKP-173,pSKP-176 and pSKP-204 constructs were generated in HEK293T/17 cells, and used to transduce HeLa cells as described before.^62^ Transgenic HeLa cells were selected in 4.5 μg/ml Blasticidin (Invivogen, ant-bl-1) and tested by western blotting for StrepHA-PPP2CA, StrepHA-PPP1CA and StrepHA-PPP2R5E expression upon 24 h 2 μg/ml doxycycline induction (Sigma-Aldrich, D9891).

### Method details

#### Phosphatase protein purification

HeLa cells expressing StrepHA-PPP1CA, StrepHA-PPP2CA and StrepHA-PPP2R5E were cultured in 10 × 15 cm dishes. 48 h prior to collection, cells were induced with 2 ng/mL doxycycline for 46 h and starved for 2 h with HBSS before harvest. Cells were lysed with modified radioimmunoprecipitation assay buffer (RIPA, 150 mM NaCl, 50 mM Tris-HCl pH = 8, 1% NP-40, 0.1% sodium deoxycholate and ethylenediaminetetraacetic acid (EDTA) free protease inhibitor (Roche, 5056489001)). Immunoprecipitations were conducted with 500 μL Strep-Tactin XT-4Flow slurry (IBA, 2-5030-010), equilibrated with lysis buffer, overnight at 4°C on a rotor. Beads were washed three times with lysis buffer and twice with washing buffer (50 mM Tris HCl pH = 7.5, 100 mM NaCl, 0.01% NP-40, 0.1 mM ethylene glycol-bis(β-aminoethyl ether)-tetraacetic acid (EGTA), 30% glycerol). Elution was carried out with 3 x 200 μL of 100 mM biotin-containing elution buffer (50 mM Tris HCl pH = 7.5, 100 mM NaCl, 2 mM dithiothreitol (DTT), 0.01% NP-40, 0.1 mM EGTA, 1 mM MnCl_2_, 30% glycerol). Samples were concentrated on a 100kD filter to 150 μL. All steps were performed at 4°C.

#### *P*-nitrophenyl phosphate activity assay

Enzyme activity for purified PPP1CA and PPP2CA complexes was tested using 5 mM *para*-nitrophenyl phosphate (pNPP) in 0.05 M Tris buffer containing 1 mM MnCl_2_ pH 8. For the time course, experimental triplicates with 5 mM pNPP were incubated at 37°C for up to 6 h with or without the inhibitor microcystein-LR (MCLR, 1 μM) with continuous measurements at 405 nm. A dilution series with 4 mM, 1.6 mM, 0.4 mM and 0.16 mM substrate in triplicates were incubated with PPP1CA or PPP2CA for 3 h followed by stopping the reaction with 0.2 M NaOH to determine enzyme activities. The extinction was measured at 405 nm. Michaelis-Menten kinetics were used to calculate enzyme activities with and without the phosphatase inhibitor okadaic acid (OA; Merck, O9381).

#### Cell lysis

For Phosphoproteome analyses cell pellets from 1 × 15 cm dishes were lysed in 8 M urea in 50 mM Tris-HCl pH 8.0. Lysates were centrifuged at 21,000 x g at 4°C for 10 min. Protein concentration was determined by BCA protein assay (Pierce, PIER23225). 300 μg total protein was used for each sample for the further workflow. For immunoblotting, cell pellets from 1 × 10 cm dishes were lysed in RIPA lysis buffer (150 mM NaCl, 50 mM Tris-HCl pH 7.6, 1% NP-40, 1% SDS, 0.1% sodium deoxycholate, benzonase 1:1000 (Speed Biosystems, YCP1200-500KU), EDTA free protease inhibitor and PhosStop (Sigma-Aldrich, 4906837001)).

#### Immunoblotting

Samples were mixed with 4x Laemmli buffer (0.24 M Tris-HCl pH 6.8, 8% sodium dodecyl sulfate (SDS), 40% glycerol, 0.1 M DTT, bromophenol blue) to 1x and incubated at 75°C for 10 min. 20 μg of total protein were separated by SDS-PAGE, transferred to a 0.45 nitrocellulose membrane (Sigma-Aldrich) using the Trans-Blot Turbo transfer for 30 min (Bio-RAD, 1704150). Membranes were blocked with 5% milk in TBS-T (10 mM Tris-HCl, 150 mM NaCl, 0.1% Tween 20) for 1 h. Primary antibody incubation was performed at 4°C overnight. Membranes were then washed three times with TBS-T for 15 min before incubation in peroxidase conjugated secondary antibodies for 2 h and washed again 3 times in TBST. Proteins were visualized with either WesternBright ECL (Advansta, K-12045-D50) or SuperSignal West Femto Chemiluminescent (Pierce, PIER34096) on the Fusion FX (Vilber). Antibodies against actin were used to quantify and normalize protein amounts. Quantification was performed using ImageJ 1.54f (Wayne Rasband and contributors, National Institutes of Health, USA).

#### Immunostainings and fluorescence microscopy analysis

For immunofluorescence analysis, the A549 cells were seeded on collagen I (ThermoFisher, A10483-01, diluted in 0.02 M acetic acid to 50 μg/mL)-coated coverslips for 24 h prior to experiments. Cells were treated as indicated and further fixed using PFA (paraformaldehyde) for 15 min. Before and after fixing, the cells were washed 6 times in 1X PBST (1X PBS, 0.1% Tween 20), and permeabilized with 0.1% Triton X-100. After fixing, cells were blocked in 1X PBST containing 5% horse serum (ThermoFisher, 16050) for 30 min, and finally washed 6 times in 1X PBST. Cells were incubated in a wet chamber with a primary antibody solution 1:100 diluted in 5% horse serum in 1X PBST, overnight at 4°C. Cells were then washed 6 times in 1X PBST and incubated in the secondary antibody solution with dilution 1:2000 in the dark, for 2 h at room temperature. After incubation, cells were washed 6 times with 1X PBST, incubated in 10 μM Hoechst 33342 solution (Sigma-Aldrich, 14533) for 1 min, washed again 6 times, and embedded in ProLong Gold antifade reagent (ThermoFisher, P36931). Confocal imaging was performed using a Leica STELLARIS 8 FALCON system. Images were analyzed, quantified, and prepared with Fiji (v.2.3.0) and Imaris (v.10.2.0, Bitplane, Oxford Instruments), employing intensity thresholding, size exclusion, and noise filtering, based on signal intensities of the control. Abnormal or dead cells were manually excluded, nuclei were detected using the surface tool, nuclei close to the edge were excluded. Surfaces (Number and volume) were detected automatically using the same parameters throughout each experiment to avoid user induced bias.

#### *In vitro* phosphatase assay

A549 cell pellets from 15 × 15 cm dishes were lysed in primary amine-free lysis buffer (50 mM HEPES pH 8.0, 1% NP-40, 150 mM NaCl, 0.1% sodium deoxycholate, 1x EDTA free protease inhibitor) and centrifuged at 3000 x g for 10 min at 4°C. The total protein amount was quantified by BCA assay, the lysate concentration was adjusted to 10 mg/mL 1 μM Microcystin LR (Lubio Science, ORB65803) and 1 mM ATP were added for 2 h at 37°C while shaking. 0.5 mL of NHS-activated Sepharose 4 fast flow beads (GE Healthcare, 17-0906-01) per sample were washed with 3 x 10 mL ice-cold 1 mM HCl and 2 x 10 mL lysis buffer prior to incubation with sample. The coupling was performed for 6 h on a rotor at 4°C. Subsequently, the beads were spun down to remove supernatant. The total protein amount and the flow-through were measured by BCA assay. Beads were then washed 3 x 10 mL with phosphatase buffer (50 mM HEPES, 100 mM NaCl, 0.1% NP-40). After resuspension in phosphatase buffer with 1 mM MnCl_2_, beads were split into tubes according to sample number. Elution buffer, lambda phosphatase and purified phosphatases were added to the negative control, positive control and the samples, respectively. For additional control over the groups 10 μM okadaic acid was added for PPP1CA and 10 nM for PPP2CA. Subsequently, the purified PPP1CA, PPP2CA and PPP2R5E underwent pre-incubation with their respective concentrations of okadaic acid (10 μM or 10 nM) for 10 min at 37°C to inhibit phosphatase activity before subsequent addition. All samples were incubated for 4 h at 37°C with shaking. Reaction tubes were frozen in liquid nitrogen and lyophilized overnight. For lysis, 8 M urea in 50 mM Tris-HCl pH 8 was added to the dry beads followed by protein digestion, solid phase extraction (SPE) purification and phosphopeptides enrichment.

#### Protein digestion and MS sample preparation

For *in vivo* and *in vitro* phosphoproteome samples, lysates or proteins on beads respectively were reduced with 1 mM DTT for 20 min, alkylated with 5.5 mM iodoacetamide (IAA) and digested with 1:100 Lys-C (Merck, US1324796) for 4 h at room temperature on a rotor. After dilution of urea concentration to 1 M with 50 mM Tris-HCl pH 8, samples were digested with 1:100 trypsin (Promega, V5113) overnight. Peptides were purified by HR-X SPE columns (Macherey-Nagel, MANA730934P45): washing with 0.1% formic acid in deionized water, elution in 80% acetonitrile and 0.1% formic acid in deionized water. Eluates were frozen in liquid nitrogen and lyophilized overnight. Samples were resuspended in 200 μL 80% acetonitrile with 0.1% trifluoroacetic acid in deionized water for phosphopeptides enrichment using Fe(III)-NTA cartridges (Agilent). Flowthrough for non-phosphopeptides analyses and phosphopeptides enriched samples were lyophilized overnight and stored at −80°C. Non phosphopeptides were desalted by STAGE tips, all samples were resuspended in 20 μL 0.1% formic acid for LC-MS/MS analysis.

#### LC-MS/MS analyses

LC-MS/MS analyses were performed on a Exploris 480 mass spectrometer coupled to an EasyLC 1200 nanoflow HPLC or a Vanquish *NEO* UHPLC system (all Thermo Scientific). Peptides were separated on a fused silica HPLC column tip (I.D. 75 μm, New Objective, self-packed with ReproSil-Pur 120 C18-AQ, 1.9 μm (Dr. Maisch) to a length of 20 cm) using a gradient of A (0.1% formic acid in water) and B (0.1% formic acid in 80% acetonitrile in water). Samples were loaded with 0% B with a flow rate of 600 nL/min; peptides were separated by 5%–30% B within 85 min with a flow rate of 250 nL/min. Spray voltage was set to 2.3 kV and the ion-transfer tube temperature to 250°C; no sheath and auxiliary gas were used. The mass spectrometer was operated in data independent mode, after each MS scan (m/z = 400–1′200; resolution: 120′000, maximum injection time of 60 ms), 34 MS/MS scans with an isolation width of 24 m/z with 1 Da overlap were performed, covering a range from 400 to 1′200 m/z. The normalized AGC target value was set to 300%, resolution to 30′000 and normalized stepped collision energy to 25.5%, 27% ND 30%. The MS raw files were analyzed using Spectronaut software version 17 or 19 and directDIA+ workflow against an UniProt full length Homo sapiens database (January 2022) and common contaminants, such as keratins, as reference. Carbamidomethylcysteine was set as fix modification and protein amino terminal acetylation, methionine oxidation and serine-, threonine- and tyrosine phosphorylation were set as variable modifications. Peptide, site and protein FDR were set to 0.01, minimum peptide length was set to 7, a maximum of two missed cleavages were allowed using trypsin/P as enzyme specificity. For protein IDs a minimum of 2 peptides had to be identified. Imputation and Multiplicity were deselected, for PTM consolidation the linear model was chosen. Each set of experimental conditions was analyzed in separate Spectronaut workflows and replicates were searched separately.

For targeted mass spectromety, LC-MS/MS measurements were performed on an EASY-nLC 1000 nano-flow UHPLC system (Thermo Fisher Scientific) coupled to a Q Exactive HF-X hybrid quadrupole-Orbitrap mass spectrometer (Thermo Fisher Scientific). Using a linear gradient of solvent A and solvent B (0.1% formic acid in 80% acetonitrile in water) from 4% solvent B to 30% over 85 min, followed by an increase to 100% buffer B over 8 min and 7 min at 100% buffer B at a 250 nL/min flow rate. The mass spectrometer was operated in PRM mode at 60,000 resolution with an AGC target set as 3e6 and a maximum injection time of 108 ms. Isolation window was set at 1.0 m/z, normalized collision energy was set to 27. Data was acquired in centroid mode. Spectral libraries were acquired using a phosphorylation enriched sample in Data Independent Acquisition with the same resolution and collision energies. The raw files were analyzed in Skyline,^61^ MS2 spectra were matched to the generated library. Extracted ion chromatograms were manually inspected, signals from interfering ions were removed and integration peak boundaries were modified if needed. Precursors with less than 3 valid transitions were excluded from further considerations. To normalize the intensity across runs, samples were normalized to the Total Ion Current (TIC). Further data analysis and data visualization was performed using R. MS raw data are available via ProteomeXchange with identifiers PXD050485 and PXD060829.^63^

### Quantification and statistical analysis

#### Quantification of proteomic data

Label free quantification was performed using Perseus 1.6.15.0.^57^ Phosphorylation sites with a maximum localization probability ≥0.75 were considered for analysis. Each condition of the phosphatase assay was merged with the control sample, followed by standard analysis workflow. Values below 5 after Log2 transformation were manually removed as those were a result of the matching across runs in Spectronaut 17/19 (Biognosys). Replacement of missing values, using the singular value decomposition (SVD) technique, was applied to phosphosites that were quantified in at least two replicates. After merging all conditions to the control, missing values in all conditions were replaced by random values of a normal distribution to mimic low abundance measurements. Width and down shift were used according to default settings for each column separately. After grouping of replicates significant changes were determined (FDR ≤0.05).

GO-term analysis was performed with Cytoscape 3.9.0^58^ and the ClueGo plugin.^59^ GO-biological processes (BP) were selected for further calculations, only pathways with an enrichment effect over 1.1 were considered. Sequence logos and motif analyses were performed using PhosphoSitePlus. The respective input sequences were chosen as background for calculations. Position specific probability matrices surrounding the phosphorylation site were calculated as described before.^28^ Sequence information in positions −7 to +7 were analyzed for all class I phosphorylation sites. For fold-change analyses in heatmaps, the relative abundance between the significantly dephosphorylated sites with a fold change of ≥3 was compared to sites without changes (−0.5 to +0.5 log2 changes). Amino acids with less than 1% counts in PPP1CA or PPP2CA sensitive/insensitive comparisons were excluded.

Plots were done in R (https://www.r-project.org/) using ggplot2,^64^ tidyverse,^65^ rstatix (https://github.com/kassambara/rstatix) and BiocManager.

#### SLiMS analysis

The phosphorylation sites modulated by PP2A were checked against previously verified sites in the PhosphositePlus database^66^ to identify newly detected phosphorylation sites. An in-house Labkey pipeline designed for searching phosphatase targets in cellular signaling and phosphoproteomic data^67^ was used to search for the previously identified PP2A B56^8^^,^^44^^,^^45^^,^^68^ and B55^35^^,^^36^^,^^37^ binding motifs. The phosphatase Labkey pipeline will be described elsewhere. In brief, it integrates bioinformatic tools, such as Find Individual Motif Occurrence (FIMO) tool^69^ from the MEME suite,^70^ streamlines SLiM sequences, data input, and searches of different motifs for all entries, and output results were integrated in a single file. In this analysis, FIMO search was used with the default *p*-value of 1e-4. Motifs were represented using the Seq2Logo service.^60^

Further analysis was conducted on the identified motifs using the tidyverse package^65^ and a customized R script (https://www.r-project.org/). The analysis aimed to classify the identified SLiMs into three categories: overlapping with, upstream and downstream of the phosphorylation sites. For B56 SLiMs, upstream SLiMs islocated at least ten residues upstream of the phosphorylation site, and downstream SLiMs at least twenty residues downstream of the phosphorylation site.

Secondary structure annotations from UniProt^71^ were used to distinguish motifs within structured regions. In addition, IUPred^72^ was used to predict the disorder propensity of motifs, and an average disorder score was calculated for each motif. Motifs with an average score of 0.5 or more were classified as disordered motifs.

## Supplemental information

Document S1. Figures S1–S3

Table S1. λ-Phosphatase-sensitive phosphosites, related to Figure 1

Table S2. PPP1CA and PPP2CA complex purifications and OBIPhA assays, related to Figure 2 (A) PPP1CA and PPP2CA complex purifications. (B) OBIPhA of PP1. (C) OBIPhA of PP2A

Table S3. LB100- and OA-sensitive sites, and bona fide PP2A target sites, related to Figure 3 (A) LB100-sensitive sites. (B) OA-sensitive sites. (C) Bona fide PP2A target sites

Table S4. PPP2R5E-PP2A complex purifications, OBIPhA assays, and bona fide target sites, related to Figure 4 (A) PPP2R5E complex purifications. (B) OBIPhA of PPP2R5E. (C) Bona fide PPP2C-PPP2R5E target sites

Document S2. Article plus supplemental information
