# Supplementary material for: Combination of in vivo and in vitro phosphoproteomics determines the PP2A target repertoire on proteome scale
Source: Cell Rep Methods. 2025 Jun 19;5(7):101084. doi: 10.1016/j.crmeth.2025.101084 (PMC12296510; doi:10.1016/j.crmeth.2025.101084)
Supplement: Document S1. Figures S1–S3 [file mmc1.pdf]

Cell Reports Methods, Volume *5*

Supplemental information

Combination of *in vivo* and *in vitro* phosphoproteomics determines the PP2A target repertoire on proteome scale

Melanie Brunner, Zehan Hu, Heidy Elkhaligy, Gloria Lampo, Carole Roubaty, Christine Vionnet, Devanarayanan Siva Sankar, Sean J. McIlwain, Stéphanie Kaeser-Pebernard, Yongna Xing, and Jörn Dengjel

Figure S1

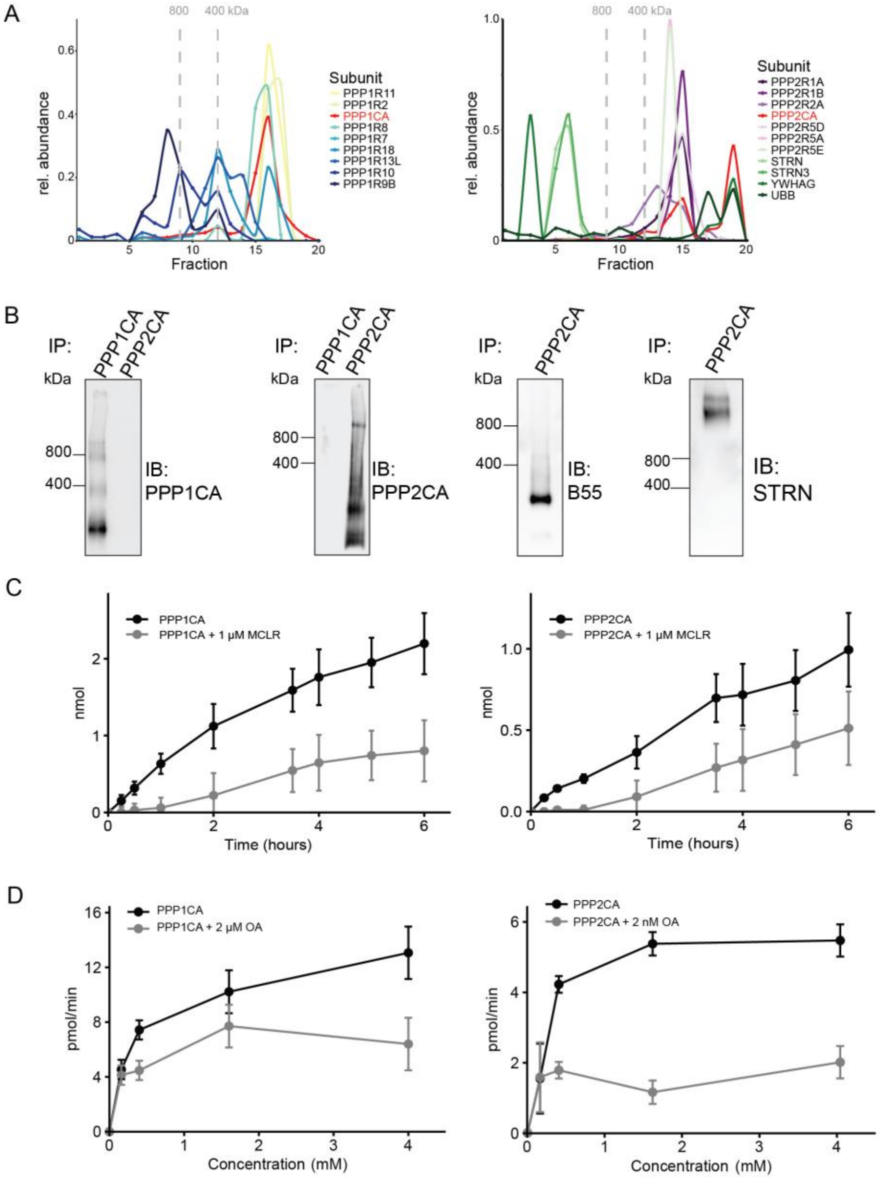

Figure S1: Complexome profiling of PP1 and PP2A complexes. Related to Figure 2. **(A)** BN-PAGE coupled to quantitative MS was performed to map protein complex compositions of PPP1CA (left) and PPP2CA (right) affinity purifications. Native protein complexes were separated by BN-PAGE, gel lanes were cut into 20 slices and proteins therein digested by trypsin prior LC-MSMS analyses. Extracted ion currents were used to determine relative abundances of indicated proteins. In both cases a complex mixture of different holo-complexes containing respective catalytic subunits were purified. **(B)** BN-PAGE coupled to western blot was performed to highlight enrichment of native complex and their purity. Shown is one representative of n=3 biological replicates. **(C-D)** Enzymatic activity assays. Purified phosphatase holocomplexes were incubated with (C) 5 mM p-Nitrophenyl phosphate (pNPP) +/- 1 μM MCLR indicating phosphatase specific and non-specific activities; and (D) increasing concentrations of pNPP to determine enzymatic activities. 2 nM okadaic acid (OA) potently inhibited PP2A, whereas 2 μM OA was needed to get similar results for PP1 complexes. Shown are average values of n=3 biological replicates, error bars indicate standard deviations.

Figure S2

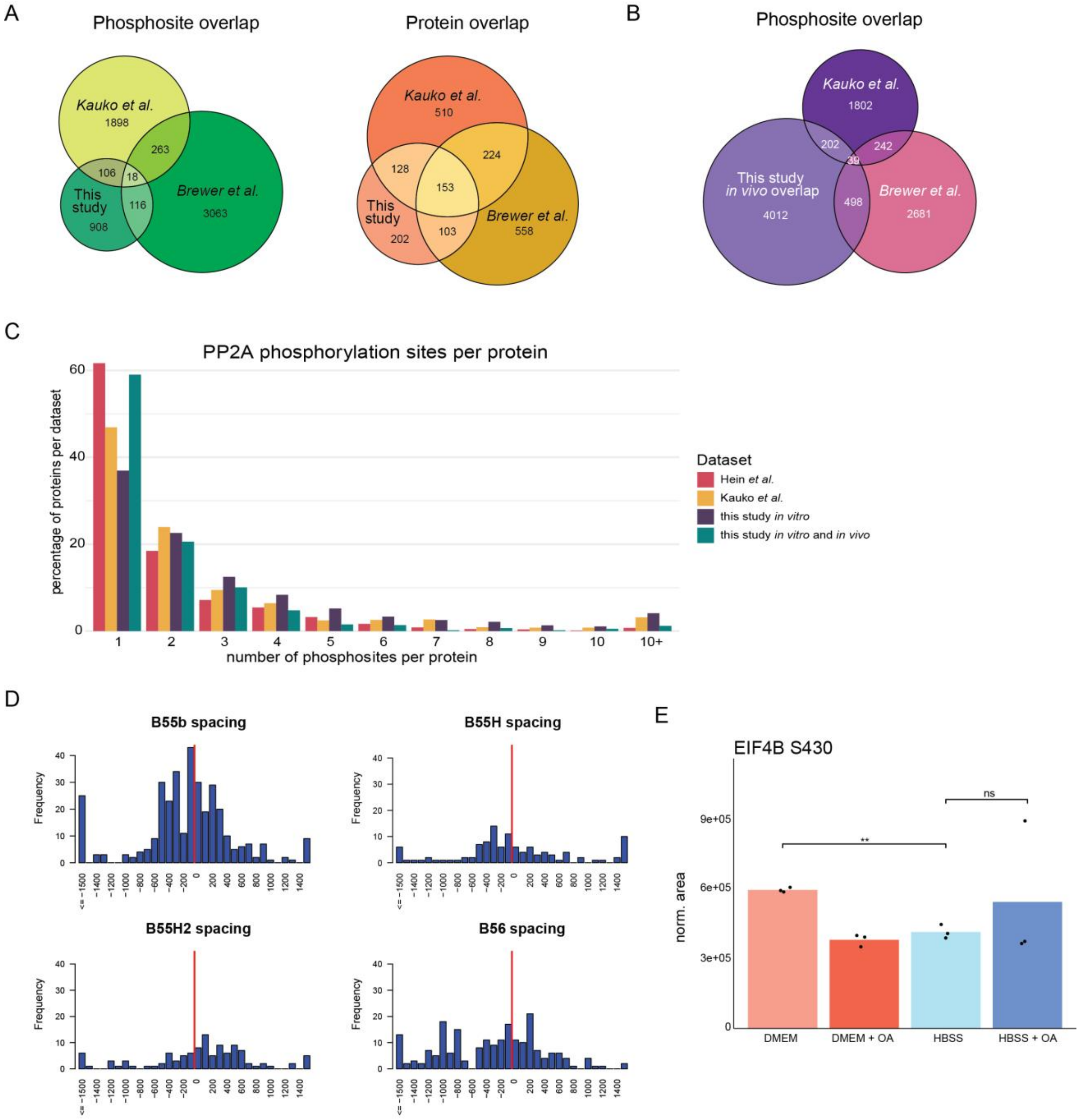

Figure S2: PP2A target sites. Related to Figure 3. **(A-B)** Phosphosite and target protein overlap of *bona fide* PP2A (A) and *in vivo* phosphoproteomic data (B) of the current study with *in vivo* data of Kauko *et al.* and Brewer *et al.* **(C)** Relative protein numbers carrying 1 or more phosphosite(s) per protein in the indicated studies. **(D)** Distribution of spacing between the identified B55 and B56 SLiMs and the *bona fide* phosphorylation sites (red lines, position 0). X axis lists lower numbers of respective amino acid bins, bin size being 100 amino acids. **(E)** Targeted phosphosites specific mass spectrometry (PRM) for EIF4B Ser430 using a singly phosphorylated peptide variant. Bar diagram shows quantification of three replicates (black dots). **: $p<0.01$, T test.

Figure S3

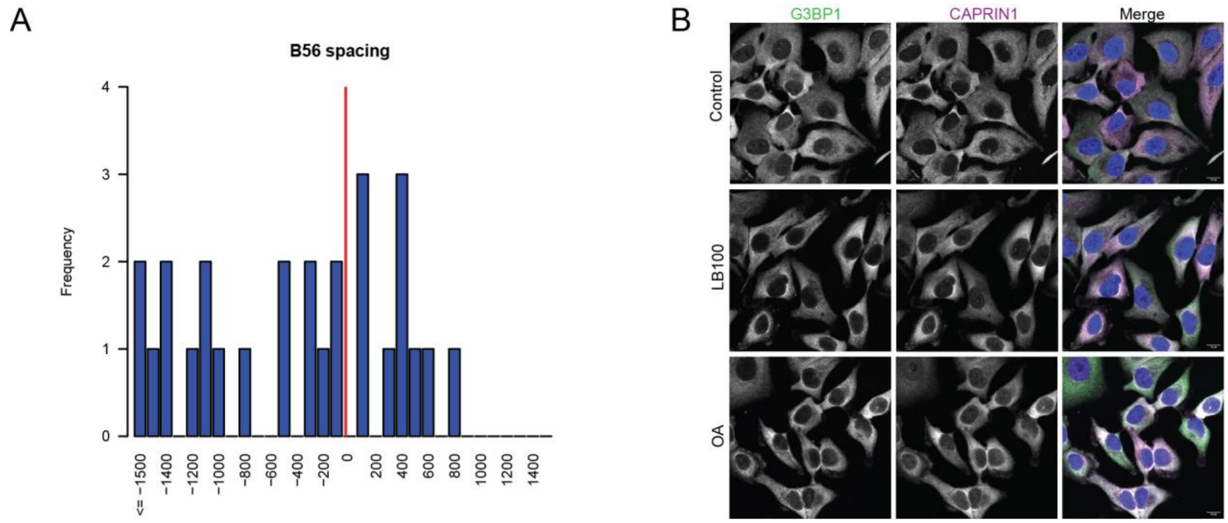

Figure S3: PPP2R5E-PP2A SLiM analysis. Related to Figure 4. **(A)** Distribution of spacing between the identified B56 SLiM and the *bona fide* phosphorylation sites (red lines, position 0). X axis lists lower numbers of respective amino acid bins, bin size being 100 amino acids. **(B)** IF of CAPRIN1 and G3BP1. Shown are exemplary images of n=3 biological replicates. Nuclei are stained in blue. Scale bar = 10 μM.
